# Supplementary material for: Impact of different mutations on Kelch13 protein levels, ART resistance, and fitness cost in Plasmodium falciparum parasites
Source: mBio. 2024 May 3;15(6):e01981-23. doi: 10.1128/mbio.01981-23 (PMC11237660; doi:10.1128/mbio.01981-23)
Supplement: Supplemental Material — Figures S1-S7, Tables S1 and S2, and descriptions of Data Sets S1-S4. [file mbio.01981-23-s0005.pdf]

Supplementary Materials for

Impact of different mutations on Kelch13 protein levels, ART resistance and fitness cost in Plasmodium falciparum parasites

Hannah M. Behrens, Sabine Schmidt, Isabelle Henshall, Patricia López-Barona, Domitille Peigney, Ricarda Sabitzki, Jürgen May, Oumou Maïga-Ascofaré, Tobias Spielmann*

*Corresponding author. Email: spielmann@bnitm.de

This PDF file includes:

Figs. S1 to S7
Tables S1 to S2
Dataset S1 to S3

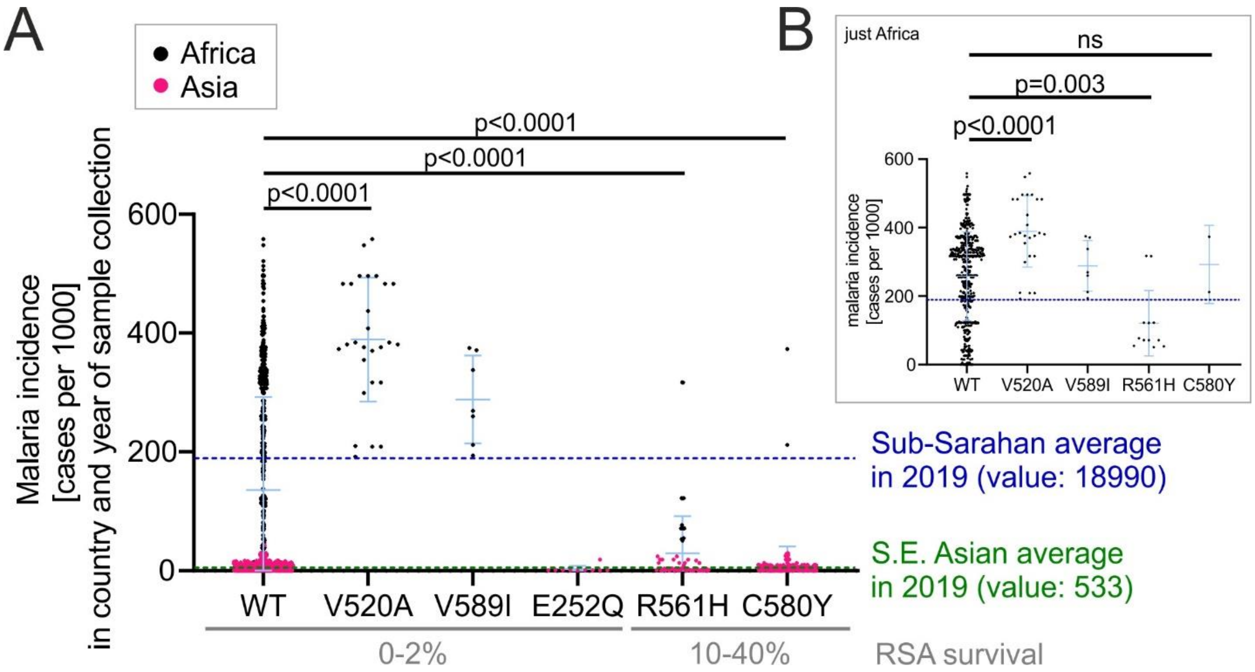

Fig. S1.

Resistance mutations R561H and C580Y are detected at below average malaria incidence. (A) Malaria incidence at place and time of *k13* mutation detection. Detection of selected k13 mutations in Africa and Asia is shown. Error bars show standard deviation. P-values from unpaired t-tests. Blue dotted line shows average malaria incidence in Sub-Saharan Africa in 2019. Green dotted line shows average malaria incidence in South East Asia in 2019. RSA survival shown as determined in this work (Figure 1B). **(B)** Same data as in A, excluding detection in Asia. P-values from unpaired t-tests of African data only.

```
K13_C580Y_sequence      -----------------------------------ATGGAGGGTGAGAAGGTTAAGACTA    25
sequencing_primer_1     CTGCTGCTGGTGCTGGAGGTGCAGGTAGACCTAGGATGGAGGGTGAGAAGGTTAAGACTA    120


K13_C580Y_sequence      AAGCTAACAGTATAAGTAACTTCAGTATGACTTACGACAGAGAGAGTGGAGGGAATAGTA    85
sequencing_primer_1     AAGCTAACAGTATAAGTAACTTCAGTATGACTTACGACAGAGAGAGTGGAGGGAATAGTA    180

K13_C580Y_sequence      ACTCAGACGACAAGAGTGGTTCAAGTTCAGAAAACGACTCAAACAGTTTTATGAACTTAA    145
sequencing_primer_1     ACTCAGACGACAAGAGTGGTTCAAGTTCAGAAAACGACTCAAACAGTTTTATGAACTTAA    240

K13_C580Y_sequence      CATCTGACAAGAACGAAAAGACAGAGAACAACTCATTTTTACTTAACAACTCATCTTACG    205
sequencing_primer_1     CATCTGACAAGAACGAAAAGACAGAGAACAACTCATTTTTACTTAACAACTCATCTTACG    300

K13_C580Y_sequence      GTAACGTAAAGGACTCTTTATTGGAGTCTATAGACATGTCAGTGTTGGACAGTAATTTCG    265
sequencing_primer_1     GTAACGTAAAGGACTCTTTATTGGAGTCTATAGACATGTCAGTGTTGGACAGTAATTTCG    360

K13_C580Y_sequence      ACTCAAAGAAGGACTTCTTGCCTTCAAACTTGTCTAGGACTTTCAACAACATGAGTAAGG    325
sequencing_primer_1     ACTCAAAGAAGGACTTCTTGCCTTCAAACTTGTCTAGGACTTTCAACAACATGAGTAAGG    420

K13_C580Y_sequence      ACAACATTGGTAACAAGTACTTGAACAAGTTATTGAACAAGAAGAAGGACACAATAACTA    385
sequencing_primer_1     ACAACATTGGTAACAAGTACTTGAACAAGTTATTGAACAAGAAGAAGGACACAATAACTA    480

K13_C580Y_sequence      ACGAGAACAACAACATAAACCACAACAACAACAACAACAACTTAACTGCTAACAACATTA    445
sequencing_primer_1     ACGAGAACAACAACATAAACCACAACAACAACAACAACAACTTAACTGCTAACAACATTA    540

K13_C580Y_sequence      CAAACAACTTAATAAACAACAACATGAACTCACCTTCTATAATGAACACAAATAAGAAGG    505
sequencing_primer_1     CAAACAACTTAATAAACAACAACATGAACTCACCTTCTATAATGAACACAAATAAGAAGG    600

K13_C580Y_sequence      AAAACTTCTTGGACGCTGCTAACTTGATTAACGACGACTCAGGTTTGAATAACTTGAAGA    565
sequencing_primer_1     AAAACTTCTTGGACGCTGCTAACTTGATTAACGACGACTCAGGTTTGAATAACTTGAAGA    660
sequencing_primer_2     ------TCTTGGACGCTGCTAACTTGATTAACGACGACTCAGGTTTGAATAACTTGAAGA    197

K13_C580Y_sequence      AGTTCTCTACAGTTAACAACGTTAACGACACATACGAGAAAAAGATAATAGAGACCGAGT    625
sequencing_primer_1     AGTTCTCTACAGTTAACAACGTTAACGACACATACGAGAAAAAGATAATAGAGACCGAGT    720
sequencing_primer_2     AGTTCTCTACAGTTAACAACGTTAACGACACATACGAGAAAAAGATAATAGAGACCGAGT    257

K13_C580Y_sequence      TGTCAGACGCATCAGACTTCGAGAACATGGTTGGAGACTTGAGGATAACTTTCATAAACT    685
sequencing_primer_1     TGTCAGACGCATCAGACTTCGAGAACATGGTTGGAGACTTGAGGATAACTTTCATAAACT    780
sequencing_primer_2     TGTCAGACGCATCAGACTTCGAGAACATGGTTGGAGACTTGAGGATAACTTTCATAAACT    317

K13_C580Y_sequence      GGTTGAAGAAAACTCAGATGAACTTCATAAGAGAGAAGGACAAGTTGTTCAAGGACAAAA    745
sequencing_primer_1     GGTTGAAGAAAACTCAGATGAACTTCATAAGAGAGAAGGACAAGTTGTTCAAGGACAAAA    840
sequencing_primer_2     GGTTGAAGAAAACTCAGATGAACTTCATAAGAGAGAAGGACAAGTTGTTCAAGGACAAAA    377

K13_C580Y_sequence      AGGAGCTTGAGATGGAGAGGGTTCGTTTATATAAGGAGTTGGAGAATCGTAAGAACATAG    805
sequencing_primer_1     AGGAGCTTGAGATGGAGAGGGTTCGTTTATATAAGGAGTTGGAGAATCGTAAGAACATAG    900
sequencing_primer_2     AGGAGCTTGAGATGGAGAGGGTTCGTTTATATAAGGAGTTGGAGAATCGTAAGAACATAG    437

K13_C580Y_sequence      AGGAGCAAAAGTTGCACGACGAGAGGAAAAAGTTGGACATAGACATTTCAAACGGATACA    865
sequencing_primer_1     AGGAGCAAAAGTTGCACGACGAGAGGAAAAAGTTGGACATAGACATTTCAAACGGATACA    960
sequencing_primer_2     AGGAGCAAAAGTTGCACGACGAGAGGAAAAAGTTGGACATAGACATTTCAAACGGATACA    497

K13_C580Y_sequence      AGCAGATTAAGAAGGAGAAGGAGGAGCACAGAAAGCGTTTCGACGAGGAGAGGTTGAGGT    925
sequencing_primer_1     AGCAGATTAAGAAGGAGAAGGAGGAGCACAGAAAGCGTTTCGACGAGGAGAGGTTGAGGT    1020
sequencing_primer_2     AGCAGATTAAGAAGGAGAAGGAGGAGCACAGAAAGCGTTTCGACGAGGAGAGGTTGAGGT    557

K13_C580Y_sequence      TCTTGCAGGAGATAGACAAGATAAAGTTGGTTTTGTACTTGGAGAAGGAGAAGTACTACC    985
sequencing_primer_1     TCTTGCAGGAGATAGACAAGATAAAGTTGGTTTTGTACTTGGAGAAGGAGAAGTACTACC    1080
sequencing_primer_2     TCTTGCAGGAGATAGACAAGATAAAGTTGGTTTTGTACTTGGAGAAGGAGAAGTACTACC    617

K13_C580Y_sequence      AGGAGTACAAGAACTTCGAAAACGACAAGAAGAAGATAGTAGACGCTAACATAGCAACAG    1045
sequencing_primer_1     AGGAGTACAAGAACTTCGAAAACGACAAGAAGAAGATAGTAGACGCTACCTAGCACCGAG    1140
sequencing_primer_2     AGGAGTACAAGAACTTCGAAAACGACAAGAAGAAGATAGTAGACGCTAACATAGCAACAG    677

K13_C580Y_sequence      AGACAATGATAGACATAAACGTAGGAGGTGCAATATTCGAGACTTCAAGGCACACTTTGA    1105
sequencing_primer_2     AGACAATGATAGACATAAACGTAGGAGGTGCAATATTCGAGACTTCAAGGCACACTTTGA    737

K13_C580Y_sequence      CTCAGCAGAAGGACTCTTTCATTGAAAAGTTGTTGTCAGGTAGGCACCACGTTACAAGGG    1165
sequencing_primer_2     CTCAGCAGAAGGACTCTTTCATTGAAAAGTTGTTGTCAGGTAGGCACCACGTTACAAGGG    797

K13_C580Y_sequence      ACAAGCAGGGTAGGATTTTTTTGGACAGAGACTCAGAATTGTTCAGGATAATTTTAAATT    1225
```

```
sequencing_primer_2       ACAAGCAGGGTAGGATTTTTTTGGACAGAGACTCAGAATTGTTCAGGATAATTTTAAATT    857

K13_C580Y_sequence        TCTTGAGGAACCCCTTGACAATTCCTATTCCTAAGGACTTGTCTGAGTCAGAGGCTTTAT    1285
sequencing_primer_2       TCTTGAGGAACCCCTTGACAATTCCTATTCCTAAGGACTTGTCTGAGTCAGAGGCTTTAT    917

K13_C580Y_sequence        TAAAGGAGGCTGAGTTCTACGGAATAAAGTTCTTGCCTTTTCCTTTGGTTTTCTGCATTG    1345
sequencing_primer_2       TAAAGGAGGCTGAGTTCTACGGAATAAAGTTCTTGCCTTTTCCTTTGGTTTTCTGCATTG    977

K13_C580Y_sequence        GAGGTTTCGACGGAGTTGAGTACTTGAACAGTATGGAGTTGTTGGACATATCACAGCAGT    1405
sequencing_primer_2       GAGGTTTCGACGGAGTTGAGTACTTGAACAGTATGGAGTTGTTGGACATATCACAGCAGT    1037

K13_C580Y_sequence        GTTGGCGAATGTGCACTCCAATGTCAACTAAGAAGGCATACTTCGGTTCTGCAGTTTTAA    1465
sequencing_primer_2       GTTGGCGAATGTGCACTCCAATGTCAACTAAGAAGGCATACTTCGGTTCTGCAGTTTTAA    1097

K13_C580Y_sequence        ACAACTTTTTGTATGTATTCGGAGGAAACAATTACGACTACAAAGCATTGTTCGAGACAG    1525
sequencing_primer_2       ACAACTTTTTGTATGTATTCGGAGGAAACAATTACGACTACAAAGCATTGTTCGAGACAG    1157

K13_C580Y_sequence        AGGTATACGACAGGTTGAGGGACGTTTGGTACGTAAGTTCAAACTTGAACATTCCAAGGA    1585
sequencing_primer_2       AGGTATACGACAGGTTGAGGGACGTTTGGTACGTAAGTTCAAACTTGAACATTCCAAGGA    1217

K13_C580Y_sequence        GGAACAACTGCGGAGTAACCTCTAACGGAAGGATATACTGCATAGGAGGTTACGACGGAT    1645
sequencing_primer_2       GGAACAACTGCGGAGTAACCTCTAACGGAAGGATATACTGCATAGGAGGTTACGACGGAT    1277
sequencing_primer_3       ------ACTGCGGAGTAACCTCTAACGGAAGGATATACTGCATAGGAGGTTACGACGGAT    72

K13_C580Y_sequence        CATCAATAATTCCCAACGTTGAGGCTTACGACCACAGAATGAAGGCTTGGGTTGAAGTAG    1705
sequencing_primer_3       CATCAATAATTCCCAACGTTGAGGCTTACGACCACAGAATGAAGGCTTGGGTTGAAGTAG    132

K13_C580Y_sequence        CTCCATTAAACACTCCAAGGTCTAGTGCAATGTATGTAGCATTCGACAACAAGATATACG    1765
sequencing_primer_3       CTCCATTAAACACTCCAAGGTCTAGTGCAATGTATGTAGCATTCGACAACAAGATATACG    192

K13_C580Y_sequence        TGATAGGAGGTACAAACGGAGAAAGGTTGAACTCAATAGAGGTTTACGAGGAGAAGATGA    1825
sequencing_primer_3       TGATAGGAGGTACAAACGGAGAAAGGTTGAACTCAATAGAGGTTTACGAGGAGAAGATGA    252

K13_C580Y_sequence        ACAAGTGGGAGCAGTTCCCTTACGCATTGTTGGAGGCAAGGTCAAGTGGTGCTGCATTCA    1885
sequencing_primer_3       ACAAGTGGGAGCAGTTCCCTTACGCATTGTTGGAGGCAAGGTCAAGTGGTGCTGCATTCA    312

K13_C580Y_sequence        ACTATTTAAACCAGATTTACGTAGTAGGTGGAATAGACAACGAGCACAATATTTTGGACT    1945
sequencing_primer_3       ACTATTTAAACCAGATTTACGTAGTAGGTGGAATAGACAACGAGCACAATATTTTGGACT    372

K13_C580Y_sequence        CTGTAGAGCAGTACCAGCCTTTCAACAAGAGGTGGCAGTTCCTTAACGGAGTTCCTGAAA    2005
sequencing_primer_3       CTGTAGAGCAGTACCAGCCTTTCAACAAGAGGTGGCAGTTCCTTAACGGAGTTCCTGAAA    432

K13_C580Y_sequence        AGAAGATGAACTTCGGTGCAGCTACTTTAAGTGACTCATACATTATAACTGGTGGTGAGA    2065
sequencing_primer_3       AGAAGATGAACTTCGGTGCAGCTACTTTAAGTGACTCATACATTATAACTGGTGGTGAGA    492

K13_C580Y_sequence        ACGGGGAGGTACTTAACTCTTGCCACTTTTTCTCACCTGACACTAACGAGTGGCAATTAG    2125
sequencing_primer_3       ACGGGGAGGTACTTAACTCTTGCCACTTTTTCTCACCTGACACTAACGAGTGGCAATTAG    552

K13_C580Y_sequence        GACCTTCATTGTTGGTACCAAGGTTCGGACATTCAGTATTGATTGCTAACATTTGA----    2181
sequencing_primer_3       GACCTTCATTGTTGGTACCAAGGTTCGGACATTCAGTATTGATTGCTAACATTTGAACCG    612
```

Fig. S2.

Sanger sequencing results of the k13 gene in K13^{C580Y}-29th parasites. CLUSTAL O(1.2.4) multiple sequence alignment.

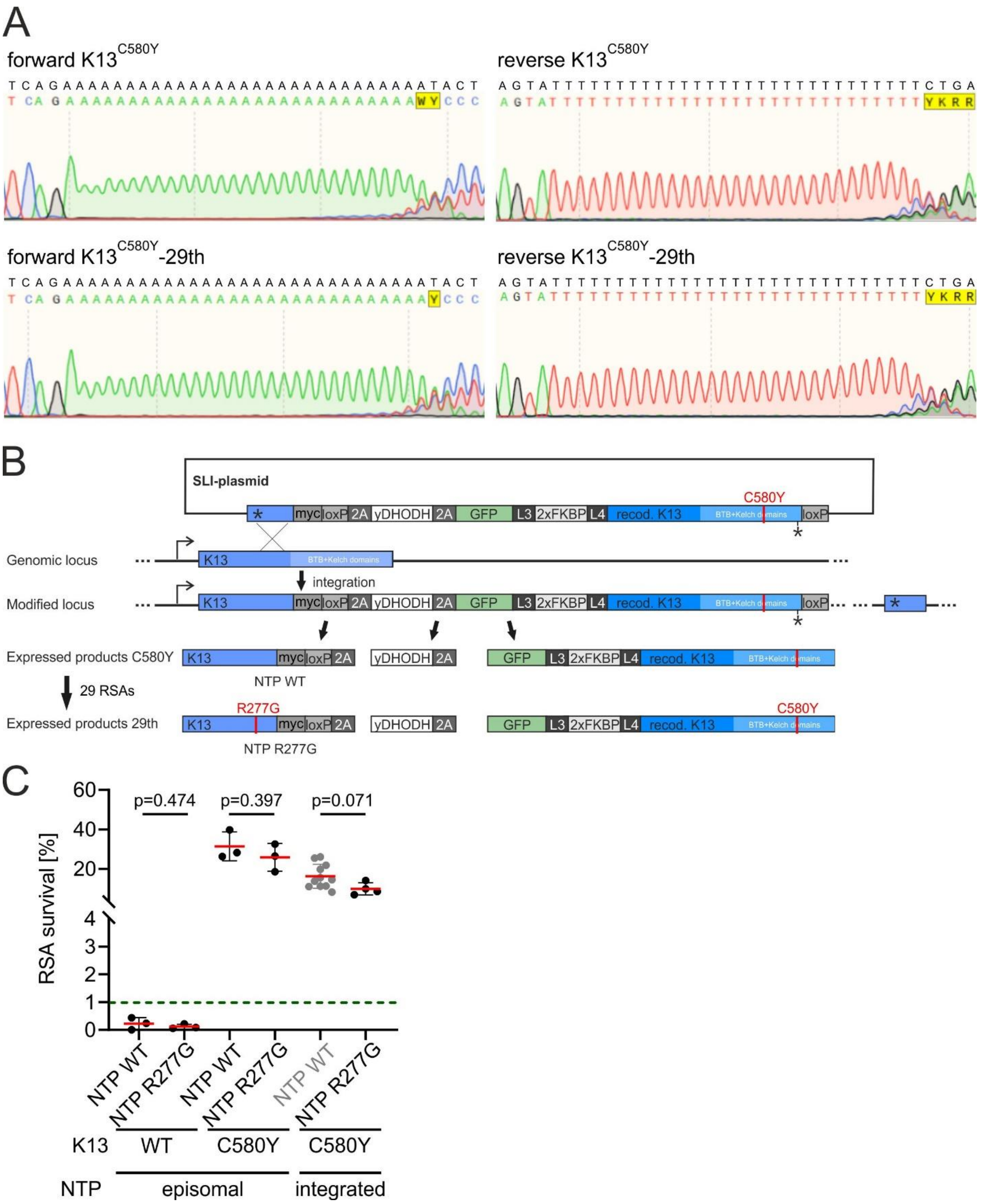

Fig. S3.

Investigation of sequence changes in *pi3k* and *k13* loci detected by whole genome sequencing. (A) Sanger sequencing of K13^{C580Y} parasites and K13^{C580Y}-29th parasites at the *pi3k* locus in forward and reverse direction. The expected sequences are shown in black, Sanger sequencing results are shown in color. **(B)** Scheme showing position of the R277G mutation in

the region of the SLI-edited *k13* locus used for homologous recombination. **(C)** ART-resistance measured by RSA of $K13^{WT}$ or $K13^{C580Y}$ parasites episomally expressing the wild type (WT) N-terminally truncated K13 part (NTP) or the NTP containing the R277G mutation, and of $K13^{C580Y}$ parasites with or without the R277G mutation in the NTP of the genomic *k13* locus. Data of $K13^{C580Y}$ parasites without the R277G mutation in the genomic NTP (grey) is reprinted from Figure 1D. p-values derive from unpaired two-tailed t-tests. Error bars show standard deviation.

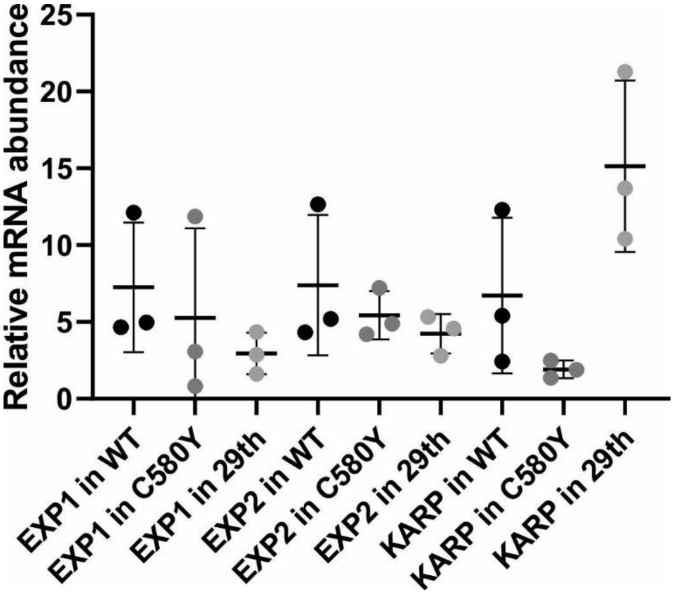

Fig. S4.

Relative mRNA abundance of k13 remain unchanged. mRNA was isolated from parasites 8 to 12 h post invasion and amplified via qPCR. Relative mRNA levels are normalized to arginyl-tRNA-synthase. For *k13* the recodonized SLI-integrated version of the gene was assessed. Data of three independent repeats is shown. P-values derive from one-way-ANOVA.

-

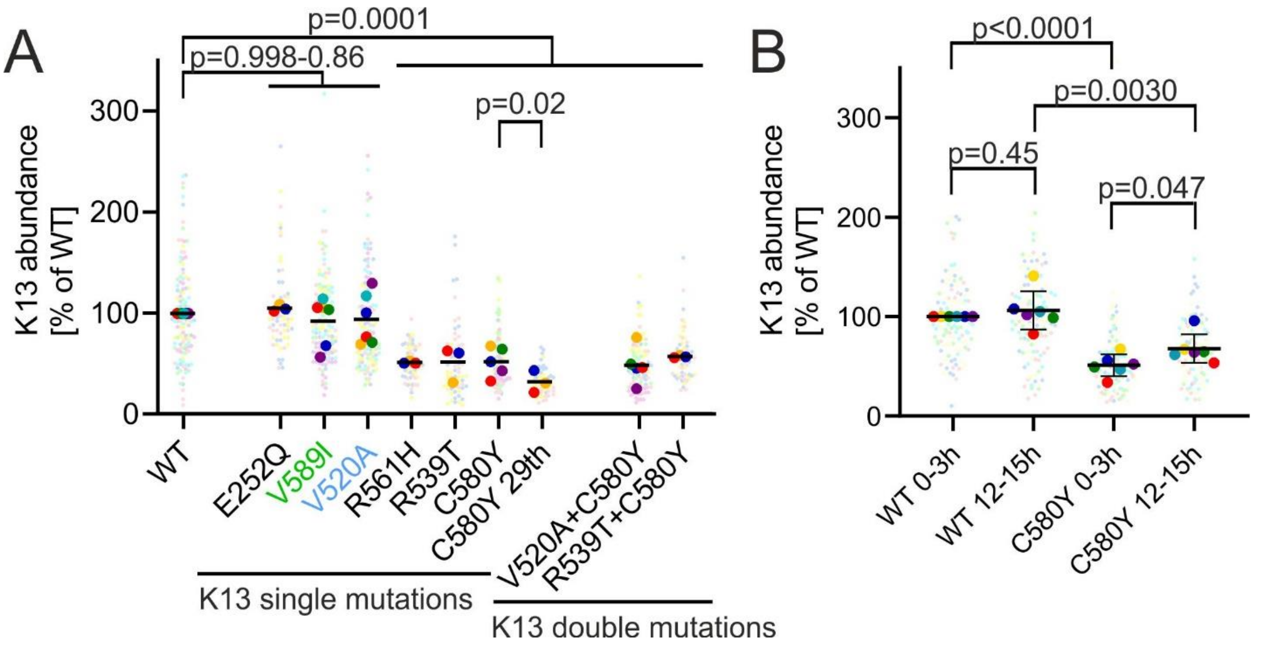

Fig. S5.

K13 quantifications. (A) K13 quantification with individual data point used for statistical analysis. K13-abundance measured by total GFP fluorescence intensity of parasites with the indicated K13 expressed from the endogenous locus and fused to GFP, normalized to the fluorescence in parasites with the identically modified endogenous locus but with a WT GFP-K13. Small dots represent measured values. Large dots of the same color as small dots represent mean of the respective measured values and each large dot represents one biological replicate. Same data as in Figure 3A. P-values derive from comparing values from each measured cell by one-way ANOVA. **(B)** K13-abundance measured by the GFP fluorescence intensity of the single focus observed in ring stage parasites of different ages. Normalized to the fluorescence in 0-3h old $K13^{WT}$ parasites. Each small dot represents the measured value from one focus in one parasite. Large dots of the same color as small dots represent mean of the respective measured values and each large dot represents one biological replicate. Each biological replicate (large dot) consists of 20 individual measurements (small dots). P-values derive from comparing means (large dots) by two-tailed unpaired t-test.

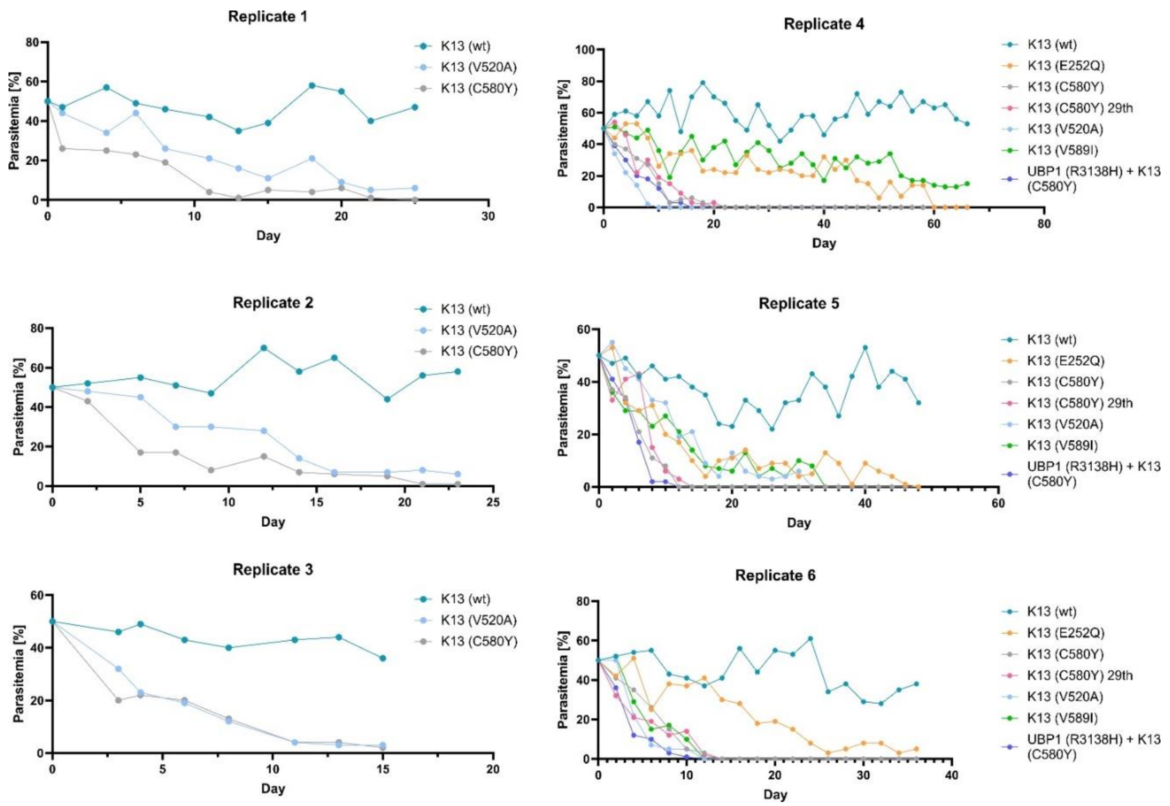

Fig. S6.

Growth curves of fitness assays. Raw curves of six independent fitness assays performed in RPMI complete medium.

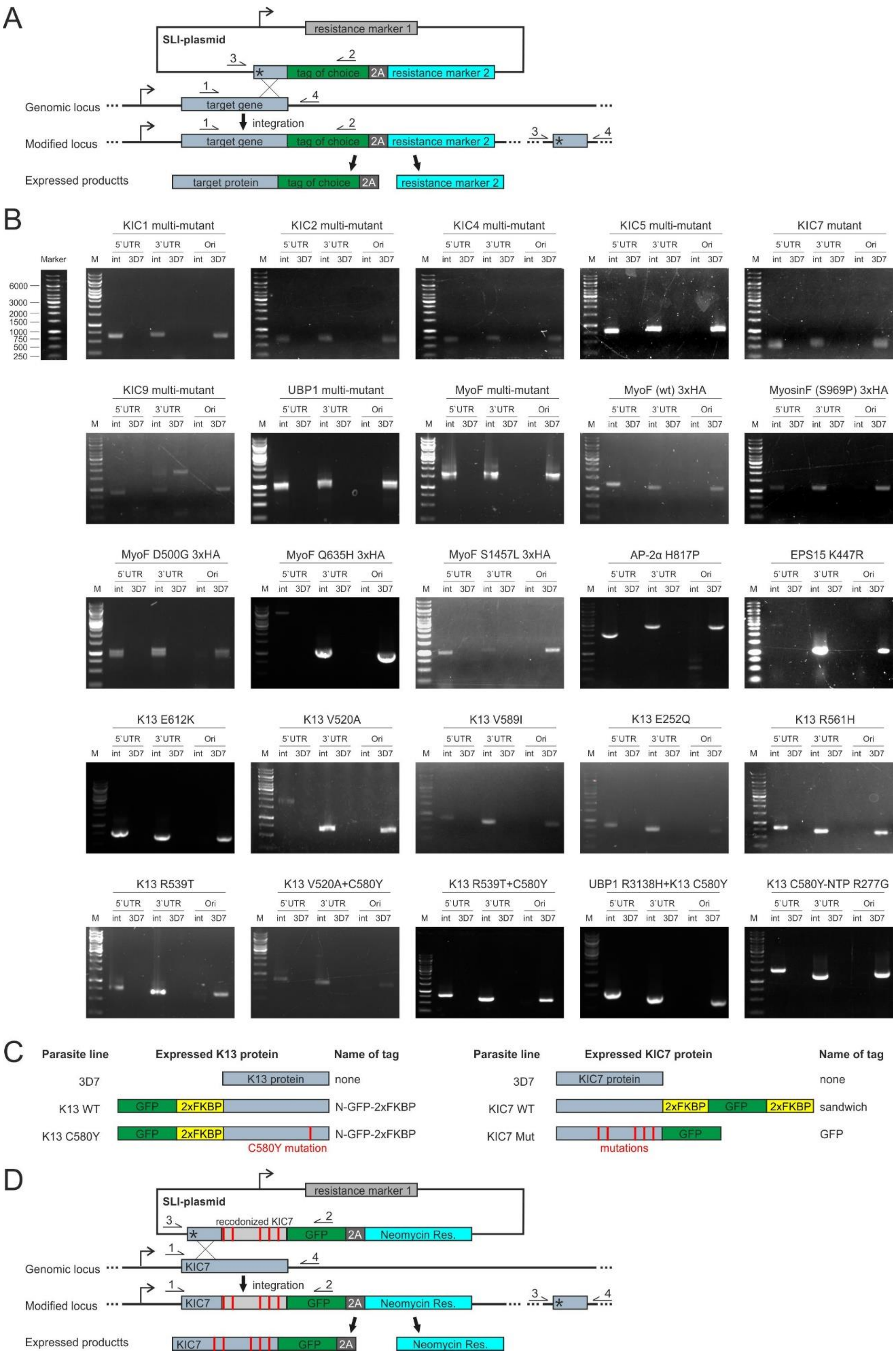
A
SLI-plasmid
resistance marker 1
3
2
tag of choice
2A
resistance marker 2
1
4
Genomic locus
target gene
integration
Modified locus
Expressed products
target protein
B
Marker
6000
3000
2000
1500
1000
750
500
250
KIC1 multi-mutant
KIC2 multi-mutant
KIC4 multi-mutant
KIC5 multi-mutant
KIC7 mutant
KIC9 multi-mutant
UBP1 multi-mutant
MyoF multi-mutant
MyoF (wt) 3xHA
MyosinF (S969P) 3xHA
MyoF D500G 3xHA
MyoF Q635H 3xHA
MyoF S1457L 3xHA
AP-2α H817P
EPS15 K447R
K13 E612K
K13 V520A
K13 V589I
K13 E252Q
K13 R561H
K13 R539T
K13 V520A+C580Y
K13 R539T+C580Y
UBP1 R3138H+K13 C580Y
K13 C580Y-NTP R277G
5'UTR
3'UTR
Ori
M
int
3D7
C
Parasite line
Expressed K13 protein
Name of tag
3D7
K13 protein
none
K13 WT
GFP
2xFKBP
N-GFP-2xFKBP
K13 C580Y
C580Y mutation
Expressed KIC7 protein
KIC7 protein
KIC7 WT
sandwich
KIC7 Mut
mutations
D
recodonized KIC7
KIC7
Neomycin Res.

Fig. S7.

Confirmation of correct integration of genome-modified parasites. (A) Schematic of selection-linked integration (SLI). **(B)** The correct integration was confirmed by generating PCR products from gDNA of indicated parasites lines, subsequently separated on agarose gels. Primers generate products across the 5' and 3' integration junctions (labelled 5' and 3') and the unmodified locus (ori), which is not present in SLI-edited lines (int) but in the parental parasites 3D7. Wild type (WT). Marker (M) fragment length is indicated in base pairs. **(C)** Schematic of expressed products in exemplarily chosen cell lines. **(D)** Schematic of selection-linked integration (SLI) to introduce mutations into genes.

Table S1.

List of all SNPs collected for multi-mutants pools and for individual testing of the candidate genes indicated. Asterisk (*), indicates SNP that were tested individually and not in the multi-mutant since they were detected after SLI plasmids of the multi-mutants were prepared for transfection or were located in the N-terminal region of the gene which would have complicated their inclusion due to the large size of this gene, and were therefore tested individually.

Candidate	Mutations
*Pf*KIC1 (PF3D7_0606000)	D483H, D535G, D862V, I873M, K231E, K676N, K883T, N179K, N633Y, N634Y, N688Y, N971S, Q1042K, S394N, S429P, T679A, V543F
*Pf*KIC2 (PF3D7_1227700)	E207Q, E343K, G167D, K321N, K651M, N449T, Y472F
*Pf*KIC4 (PF3D7_1246300)	D120N, I121V, M375V, N123S, N405S, N624I, N661H, P170S, P621A, T888A, V619A, Y179C
*Pf*KIC5 (PF3D7_1138700)	A471T, D169N, E1055G, E1354D, E1646G, E497Q, F570L, G1417D, G264D, G727E, H1198Y, H138Y, K1411R, K258R, K384I, K751Q, K985N, L1066P, M324V, M975I, N1268H, N525S, P516S, Q110E, Q1127K, Q1421K, R1040K, S1044R, S1721A, T1381A, V348I, V72I, V836I
*Pf*KIC7 (PF3D7_0813000)	I130V, N434S, N596K, P318S, S576N
*Pf*KIC9 (PF3D7_1442400)	D1345N, D1879V, D2018E, D388E, D734Y, E1861K, G674V, G984R, H1710R, I1280L, I1688V, I429S, I960V, K1039N, K1626N, K589R, K837I, L279I, M1507I, M618T, N1402K, N286H, N927H, P249Q, Q1045E, S1549N, S282N, S341N, S753T, T990R, T996I, V1586I, V2072M, Y293N
*Pf*MyosinF (PF3D7_1329100)	D500G*, Q635H*, S969P*, S1457L*, C1196S, G873D, H1587R, I2068T, L1165V, M1872L, N1615K, V1193E
*Pf*UBP1 (PF3D7_0104300)	D2704E, D2925N, G2810C, K3013Q, M2618V, N2165I, N2669S, Q2355E, Y2530H
*Pf*Eps15 (PF3D7_1025000)	K447R*
*Pf*AP-2α (PF3D7_0617100)	H817P*

Table S2.
Parasite lines used in this study.

Parasite line	Tag	Source	Parental parasite line
3D7	none	Walliker et al., 1987	NF54
KIC1 WT	Sandwich	Birnbaum et al., 2020	3D7
KIC1 Mut	GFP	This study	3D7
KIC2 WT	Sandwich	Birnbaum et al., 2020	3D7
KIC2 Mut	GFP	This study	3D7
KIC4 WT	Sandwich	Birnbaum et al., 2020	3D7
KIC4 Mut	GFP	This study	3D7
KIC5 WT	Sandwich	Birnbaum et al., 2020	3D7
KIC5 Mut	GFP	This study	3D7
KIC7 WT	Sandwich	Birnbaum et al., 2020	3D7
KIC7 Mut	GFP	This study	3D7
KIC9 WT	Sandwich	Birnbaum et al., 2020	3D7
KIC9 Mut	GFP	This study	3D7
UBP1 WT	2xFKBP-GFP	Birnbaum et al., 2020	3D7
UBP1 Mut	GFP	This study	3D7
MyoF Mut	GFP	This study	3D7
MyoF WT 3xHA	3xHA	This study	3D7
$MyoF^{S969P}$	3xHA	This study	3D7
$MyoF^{D500G}$	3xHA	This study	3D7
$MyoF^{Q635H}$	3xHA	This study	3D7
$MyoF^{S1457L}$	3xHA	This study	3D7
AP-2α^{H817P}	3xHA	This study	3D7
$EPS15^{K447R}$	GFP	This study	3D7
K13 WT	N-GFP-2xFKBP	Birnbaum et al., 2017	3D7
$K13^{E612K}$	N-GFP-2xFKBP	This study	3D7
$K13^{V520A}$	N-GFP-2xFKBP	This study	3D7
$K13^{V589I}$	N-GFP-2xFKBP	This study	3D7
$K13^{E252Q}$	N-GFP-2xFKBP	This study	3D7
$K13^{C580Y}$	N-GFP-2xFKBP	Birnbaum et al., 2017	3D7
$K13^{R561H}$	N-GFP-2xFKBP	This study	3D7
$K13^{R539T}$	N-GFP-2xFKBP	This study	3D7
$K13^{V520A+C580Y}$	N-GFP-2xFKBP	This study	3D7
$K13^{R539T+C580Y}$	N-GFP-2xFKBP	This study	3D7
$K13^{C580Y}$-29th	N-GFP-2xFKBP	This study	$K13^{C580Y}$
K13 WT +K13-NTP-WT	N-GFP-2xFKBP	This study	K13 WT
K13 WT +K13-NTP^{R277G}	N-GFP-2xFKBP	This study	K13 WT
K13 C580Y +K13-NTP-WT	N-GFP-2xFKBP	This study	$K13^{C580Y}$
K13 WT +K13-NTP^{R277G}	N-GFP-2xFKBP	This study	$K13^{C580Y}$
$K13^{C580Y}$-NTP^{R277G}	N-GFP-2xFKBP	This study	3D7
K13 1xNLS	N-GFP-2xFKBP + mCherry	Birnbaum et al., 2020	K13 WT
KIC7 1xNLS	N-GFP-2xFKBP + mCherry	Birnbaum et al., 2020	KIC7 WT
$UBP1^{R3138H}$	3xHA	Birnbaum et al., 2020	3D7
$UBP1^{R3138H}$ +$K13^{C580Y}$	3xHA + N-GFP-2xFKBP	This study	$UBP1^{R3138H}$

Dataset S1 (separate file).
List of *k13* V520A, V589I and E612K mutations found in Africa. Data were extracted from WWARN Artemisinin Molecular Surveyor database.

Dataset S2 (separate file).
Source data for Figure 4E. Malaria incidence at place and time of *k13* resistance mutation detection.

Dataset S3 (separate file).
Whole genome sequencing results of $K13^{C580Y}$ and $K13^{C580Y}$-29th parasites.

Dataset S4 (separate file).
Primers used in this study.
